# National burden of the pharmaceutical cost of wet compresses and its cost predictors: nationwide cross-sectional study in Japan

**DOI:** 10.1186/s13561-019-0238-6

**Published:** 2019-06-29

**Authors:** Hiroaki Itoh, Tomoyuki Saito, Shuko Nojiri, Yoshimune Hiratsuka, Kazuhito Yokoyama

**Affiliations:** 1 Department of Epidemiology and Environmental Health, Juntendo University Faculty of Medicine, 2-1-1 Hongo, Bunkyo-ku, Tokyo, 113-8421 Japan; 2 Department of Cardiovascular Medicine, Juntendo University Graduate School of Medicine, 2-1-1 Hongo, Bunkyo-ku, Tokyo, 113-8421 Japan; 3 Department of Pharmacy, Juntendo University Hospital, 3-1-3 Hongo, Bunkyo-ku, Tokyo, 113-8431 Japan; 4 Medical Technology Innovation Center, Juntendo University, 2-1-1 Hongo, Bunkyo-ku, Tokyo, 113-8421 Japan; 5 Department of Ophthalmology, Juntendo University Faculty of Medicine, 2-1-1 Hongo, Bunkyo-ku, Tokyo, 113-8421 Japan; 6 Department of Epidemiology and Social Medicine, International University of Health and Welfare Graduate School of Public Health, 4-1-26 Akasaka, Minato City, Tokyo, 107-8402 Japan

**Keywords:** National database, Health insurance claims, Receipt, Wet compress, Poultice, Cost

## Abstract

**Background:**

Although a high number of wet compresses are prescribed daily in medical institutions in Japan, our understanding of the national burden of the cost of wet compresses and the details regarding their prescription is far from complete. We investigated the national burden of the annual pharmaceutical cost of wet compresses prescribed in Japan and estimated the predictors of this cost using nationwide health insurance claims data.

**Methods:**

We extracted the records on wet compress products from summary table files obtained from the second version of the “NDB Open Data Japan” website and calculated the annual pharmaceutical cost of wet compresses by patients’ 5-year age group, sex, and prefecture. We also conducted an ecological study treating each prefecture as an individual unit and multiple linear regression analyses using the age-standardized cost of wet compresses per resident as a dependent variable.

**Results:**

The annual pharmaceutical cost of wet compresses prescribed in Japan in fiscal year 2015 was 149.0 billion Japanese yen (1.18 billion euros; 1.33 billion USD). Multiple linear regression analyses showed that the number of orthopedists and rehabilitation physicians per 100,000 residents were significantly positively associated with the annual pharmaceutical cost of wet compresses per resident (*P* = 0.042 and *P* = 0.008, respectively).

**Conclusions:**

The annual pharmaceutical cost of wet compresses prescribed in Japan has a considerable impact on the nation’s limited healthcare resources. The number of orthopedists and rehabilitation physicians per 100,000 residents may be independent predictors of the wet compress cost in Japan.

## Background

Japan has a super-aging society, and as such it is necessary to control increasing medical expenses. Although the Japanese government has reported that drug expenses represent just over 20% of all medical expenses, the exact total of drug expenses for the whole country is unclear because of a lack of aggregated statistics [1]. Thus, countermeasures based on statistical data are essential to eliminate unnecessary medical treatments and to encourage the efficiency and sustainability of universal health coverage in Japan. This also raises the issue of how medical care is provided in Japan [2]. Quantitative information will clarify the actual state of Japan’s medical environment. One outstanding issue to be determined is the cost of compresses in Japan [3].

A wet compress (an adhesive agent, also known as a cataplasm or poultice) is a pad or cloth made of an absorbent material that is pressed on to the body to relieve inflammation or pain. In Japan, a high number of wet compresses are prescribed daily in medical institutions (e.g., for the symptomatic treatment of knee osteoarthritis or lower back pain) [4], and they are readily available as over-the-counter purchases. Despite being a high-use product, our understanding is far from complete in terms of the national economic burden of wet compresses prescribed in Japan and their distribution patterns and predictors. Osteoarthritis is the most prevalent chronic rheumatic disease and is a leading cause of pain and disability in most countries worldwide [5]; its incidence is also rapidly increasing with population aging [6]. The total medication cost of osteoarthritis in France in 2002 was estimated to be 570 million euros (72.0 billion Japanese yen (JPY) and 0.642 billion USD, where 1 USD = 0.8874 euros = 112.166 JPY [7]) [8]. This amount represents one-third of the total direct cost of osteoarthritis in France for that year. The direct cost accounted for approximately 1.7% of the total expenses of the French health insurance system, leading to the conclusion that osteoarthritis is a major public health burden [8]. Regarding this issue, other countries with aging populations, such as Japan, cannot afford to be mere bystanders on this issue. Moreover, in the United States, 185.5 billion USD in annual out-of-pocket and insurer expenditures during 1996–2005 were attributable to medical care for patients with osteoarthritis [9]. The total pharmaceutical cost of wet compresses prescribed in Japan may be placing pressure on national healthcare financing and therefore should not be ignored. To develop effective countermeasures to facilitate the sustainability of the healthcare system, accurate figures and detailed analyses are required.

In 2009, the National Database of Health Insurance Claims and Specific Health Checkups of Japan (NDB) was developed by the Japanese government to cover almost all health insurance schemes [10, 11]. The database accumulates health insurance claims every month and specific health checkup data every year, resulting in one of the most exhaustive national-level healthcare databases in the world [12]. This nationwide insurance claims database is highly comprehensive because Japanese citizens are generally covered by employer- or community-based social health insurance [13]. Using this universal health coverage, the NDB archives almost all information listed in health insurance claims to assess the present situation of the medical environment in Japan [2, 10]. The statistics are based on claims relating to outpatients, inpatients, Diagnosis Procedure Combination (DPC) inpatients, prescriptions, dental treatments, and specific health checkups [12]. The NDB has been used as secondary data for research purposes since 2011 [2].

In addition to the NDB, the Ministry of Health, Labour and Welfare of Japan also published online the “NDB Open Data Japan” in 2016 [14] and 2017 [15], providing various summary tables from the NDB. These are fundamental spreadsheets and are freely available for use by the general public [12]. Although the “NDB Open Data Japan” does not contain data on individual patients and diseases, the annually prescribed numbers of pharmaceutical products are available.

In the present study, we investigated the national burden of the pharmaceutical cost of wet compresses prescribed in Japan and estimated the predictors of this cost using the second version of the “NDB Open Data Japan,” data covering nationwide health insurance claims in Japan.

## Methods

### Data source and calculation of wet compress costs

The authors obtained data on the number of each wet compress product prescribed annually and the corresponding pharmaceutical prices from the second version of the “NDB Open Data Japan” website [15] described above. The collection period for these data was from April 1, 2015 to March 31, 2016. There were two spreadsheet files regarding prescribed medicines for external application: one included the number of treatments prescribed and the price by sex and 5-year age groups [16], and the other was categorized by prefecture (for the 47 prefectures in Japan) [17]. Using the first of these files [16], we extracted the records on pharmaceutical products based on their pharmaceutical names, including “*shippu*” (wet compress), “tape,” or “pap” (cataplasm; poultice), in the category of “analgesic, antipruritic, astringent, or antiphlogistic” prescribed in-hospital or out-of-hospital to outpatients or inpatients [3]. The annual pharmaceutical costs of wet compresses were then calculated using eq. (1).

1 $$ \mathrm{Annual}\ \mathrm{pharmaceutical}\ \mathrm{cost}\ \mathrm{of}\ \mathrm{wet}\ \mathrm{compresses}=\sum \limits_{i=1}^n{P}_i\times {Q}_{i.} $$

Here, *P*_*i*_ and *Q*_*i*_ indicate the drug price and the annual number of prescriptions for a wet compress product (*i*), respectively. The total number of wet compress products prescribed to outpatients or inpatients is *n*. This calculation was used to calculate the pharmaceutical cost separately by a patient’s sex and 5-year age group.

Using the second spreadsheet file described above [17], we also performed an ecological study that treated each prefecture as an individual unit (*n* = 47). Again, we used eq. (1) to calculate the annual pharmaceutical cost of wet compresses by prefecture.

### Statistical analysis

In the ecological study, multiple linear regression analysis was performed. The dependent variable was the annual pharmaceutical cost of wet compresses per resident, and this was adjusted for age using indirect standardization. In this calculation, people aged 85 years or older were included in one category. Independent variables were the number of rheumatology physicians per 100,000 residents in 2014 (when the number of physicians was counted, multiple medical specialties were allowed) [18], the population sex ratio in 2015 [19], and the log_10_-transformed annual per capita income of residents in 2014 [20]. The population sex ratio was adjusted for age using direct standardization. In other models, the number of orthopedists, rehabilitation physicians, pediatricians, and internists [18] were used as independent variables (rather than the number of rheumatology physicians). Accordingly, five multiple linear regression models were individually estimated using the REG procedure in SAS version 9.4 for Windows (SAS Institute, Cary, NC, USA). The variance inflation factors ranged from 1.02 to 2.47, indicating that multicollinearity was not a problem. All *P*-values were two-sided, and *P* < 0.05 was considered statistically significant.

An intensity map showing the regional variation (quintiles) in the age-standardized wet compress cost per resident was also drawn by prefecture using an Excel-based mapping tool (www.sinfonica.or.jp) [21].

## Results

**Fig. 1:**
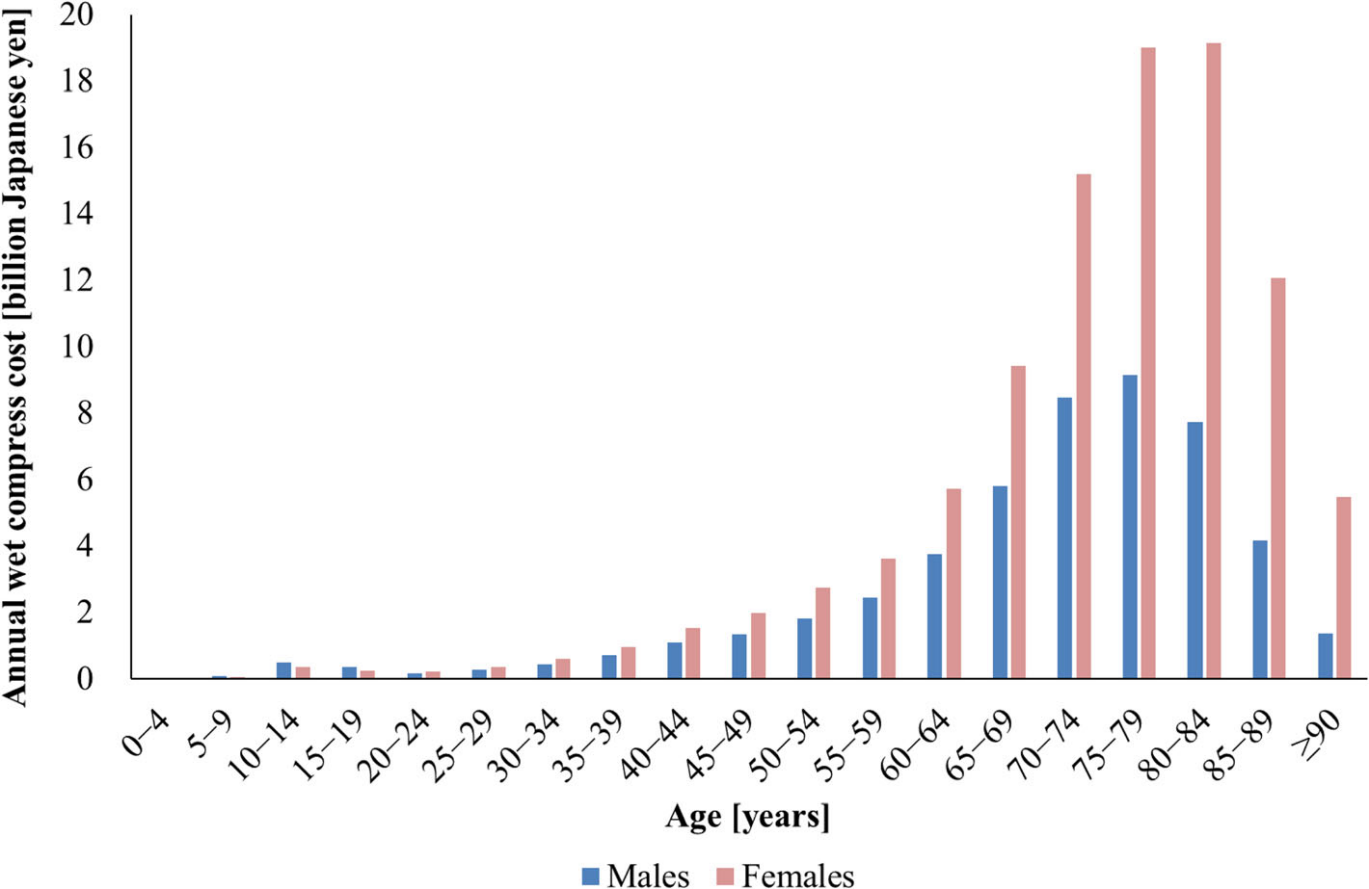
Distribution of annual wet compress cost by sex and five-year age group

Figure 1 shows the distribution of the annual pharmaceutical cost of wet compresses by sex and 5-year age group. The total annual pharmaceutical cost of wet compresses in Japan in fiscal year 2015 was 149.0 billion JPY (1.18 billion euros; 1.33 billion USD). The total cost of wet compresses prescribed to female patients was approximately two times the cost of those prescribed to male patients. People aged 65 years or older accounted for 79% of the annual total cost of wet compresses (117 billion JPY). Generic products made up 11.8% of the total wet compress cost in Japan.

**Fig. 2:**
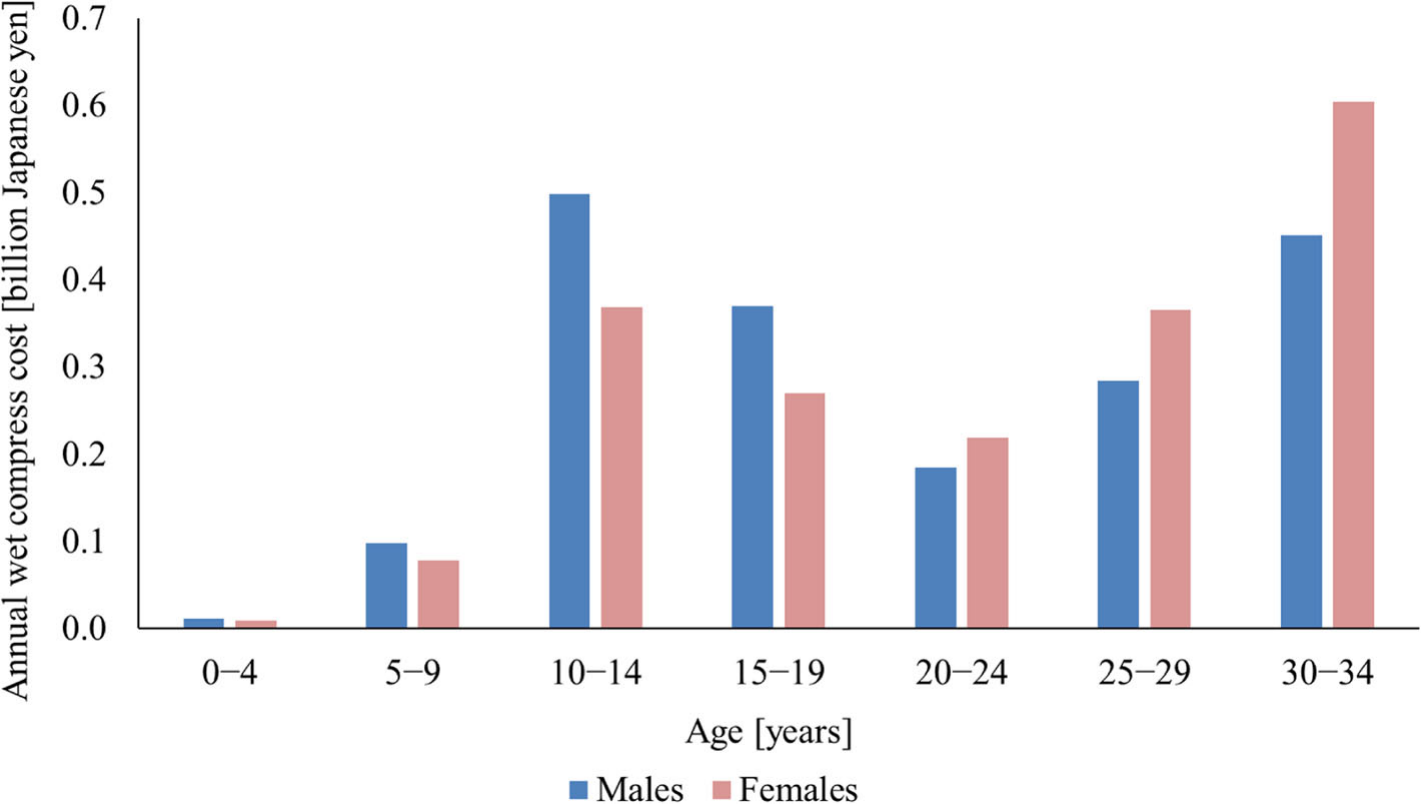
Distribution of annual wet compress cost by sex and five-year age group among younger people

Figure 2 shows the results of a tail analysis focusing on the younger age groups. Among people aged younger than 20 years, the annual pharmaceutical costs of wet compresses prescribed to male patients were consistently higher than the costs prescribed to female patients.

Table 1 shows the results of the multiple linear regression analyses in the ecological study. The number of orthopedists and rehabilitation physicians per 100,000 residents were significantly positively associated with the annual pharmaceutical costs of wet compresses (*P* = 0.042 and *P* = 0.008, respectively). There was no significant association between the wet compress cost and the population sex ratio or residents’ income (data not shown). The number of rheumatology physicians, internists, and pediatricians were not significantly associated with the wet compress cost, and the association between the number of rheumatology physicians and the wet compress cost was borderline significant (*P* = 0.074).

**Table 1:** Associations between several factors and the annual pharmaceutical cost of wet compresses per resident: Results from five independent multiple linear regression analyses in the ecological study using each prefecture as an individual unit (*n* = 47)

Independent variable	Beta (95% CI)	*P*
Number of rheumatology physicians per 100,000 residents	28.2 (−2.8, 59.2)	0.074
Number of orthopedists per 100,000 residents	**17.1 (0.62, 33.6)**	**0.042**
Number of rehabilitation physicians per 100,000 residents	**15.4 (4.2, 26.6)**	**0.008**
Number of pediatricians per 100,000 residents	7.0 (−3.7, 17.7)	0.20
Number of internists per 100,000 residents	2.8 (−1.7, 7.2)	0.21

The dependent variable was the age-standardized annual pharmaceutical cost of wet compresses per resident

Beta indicates partial regression coefficients adjusted for covariates (age-standardized population sex ratio and annual income per resident) in each multiple linear regression model

Bold text indicates statistically significant associations (*P* < 0.05)

Adjusted R^2^ ranged from 0.180 to 0.278

*CI* Confidence interval

**Fig. 3:**
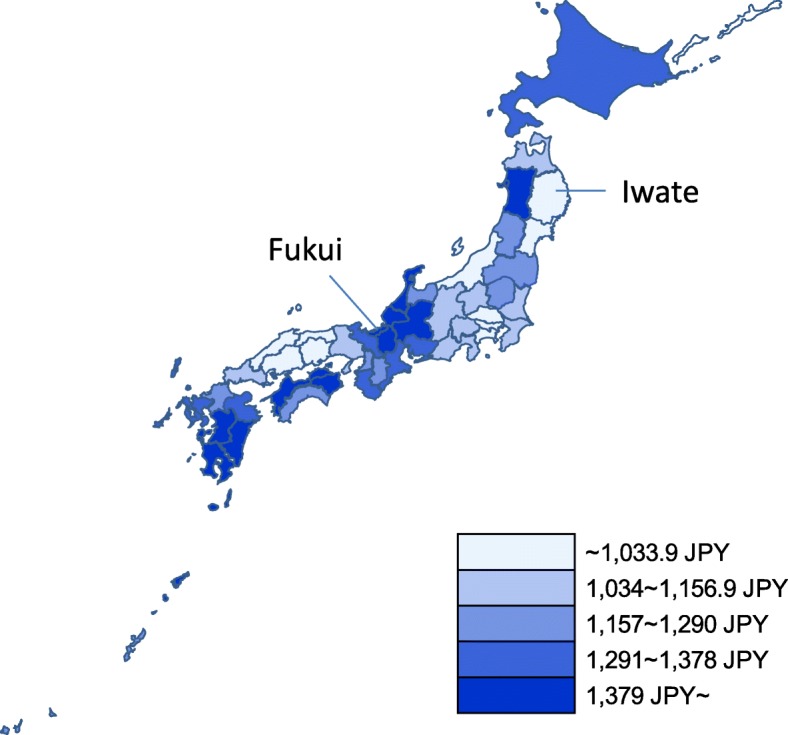
Intensity map showing the regional variation in age-standardized wet compress cost per resident (quintiles, JPY)

From a descriptive point of view, the annual pharmaceutical cost of wet compresses per resident was lowest in Kanagawa Prefecture and highest in Tokushima Prefecture. The wet compress cost in Tokushima was nearly twice that in Kanagawa. The age-standardized wet compress cost per resident was lowest in Iwate Prefecture (838 JPY) and highest in Fukui Prefecture (1613 JPY) as shown in Fig. 3.

## Discussion

The annual pharmaceutical cost of wet compresses prescribed in Japan was calculated to be 149.0 billion JPY (1.18 billion euros; 1.33 billion USD). While this result would seem reasonable at first glance, it is too high in light of Japan’s restricted healthcare financial conditions. To our knowledge, the present study is the first to provide this information. This cost is twice the estimated overall medication cost of osteoarthritis in France in 2002 (570 million euros) [22] and is comparable to the cost of the novel and extremely expensive anticancer drug Opdivo (nivolumab, 118.9 billion JPY) in Japan [23]. The drug price of Opdivo in Japan was hastily halved to reduce a sudden increase in the national healthcare costs in 2017. The wet compress cost per capita in Japan in 2015 was calculated to be 1172 JPY (9.28 euros; 10.45 USD). This is similar to the overall medication cost of osteoarthritis per capita in France in 2002 (9.22 euros). Thus, the national expense for wet compresses in Japan could be described as excessive.

The higher cost of wet compresses prescribed to older adult women is consistent with their higher prevalence of knee osteoarthritis [24–27]. Women are more likely than men to suffer from knee osteoarthritis because of women’s lower quadriceps strength and higher degree of knee joint laxity [24, 28]. Quadriceps strengthening exercises and weight control are recommended to decrease the risk of knee osteoarthritis [24]. The promotion of such preventive measures might also be effective to some extent in reducing wet compress costs. Occupations requiring heavy lifting, kneeling, or squatting and a history of knee surgery are also considered as risk factors for knee osteoarthritis [5]. Wet compress costs were lower among people aged 85 years and over than among people aged 80–84 years because of the smaller number of survivors in the older group. Additionally, the co-payment rate for people aged 75 years or older with incomes below those of average workers was only 10%, which may have contributed to the high cost of wet compresses prescribed to people in this age group. At the lowest estimate, 4.79 billion wet compresses were prescribed in Japan in fiscal year 2015, and the total number of the patients with disorders of the musculoskeletal system or connective tissue in the country in fiscal year 2014 was 5.279 million [29]. Therefore, an average of at least 908 wet compresses may have been prescribed per patient in Japan in these years. Incidentally, unused medicine generated from excess supply is a problem in Japan. Since April 2016, the number of wet compresses that can be prescribed in the country has been restricted to 70 or fewer per outpatient visit, as a general rule [30].

Possible medical alternatives to wet compresses also warrant mention. Multidisciplinary treatment including oral analgesics is effective for treating chronic pain, according to a clinical practice guideline for chronic pain [31]. Some guidelines recommend core treatments for knee osteoarthritis, such as exercise, education, weight control, and self-management [32]. An intervention study in the United Kingdom showed that the mean outpatient physiotherapy intervention cost for knee pain was 130 euros per person [33]. This study also showed that a rehabilitation program integrating exercise and self-management (mean = 64 euros) was more cost-effective than physiotherapy. Applying physiotherapy intervention and a rehabilitation program integrating exercise and self-management for all eight million symptomatic patients with knee osteoarthritis in Japan [32] would cost 1.04 billion and 512 million euros (131 billion and 64.7 billion JPY), respectively, according to the highest estimates. However, in practice, not all of these symptomatic patients with knee osteoarthritis in Japan would actually visit hospitals or clinics.

**Fig. 4:**
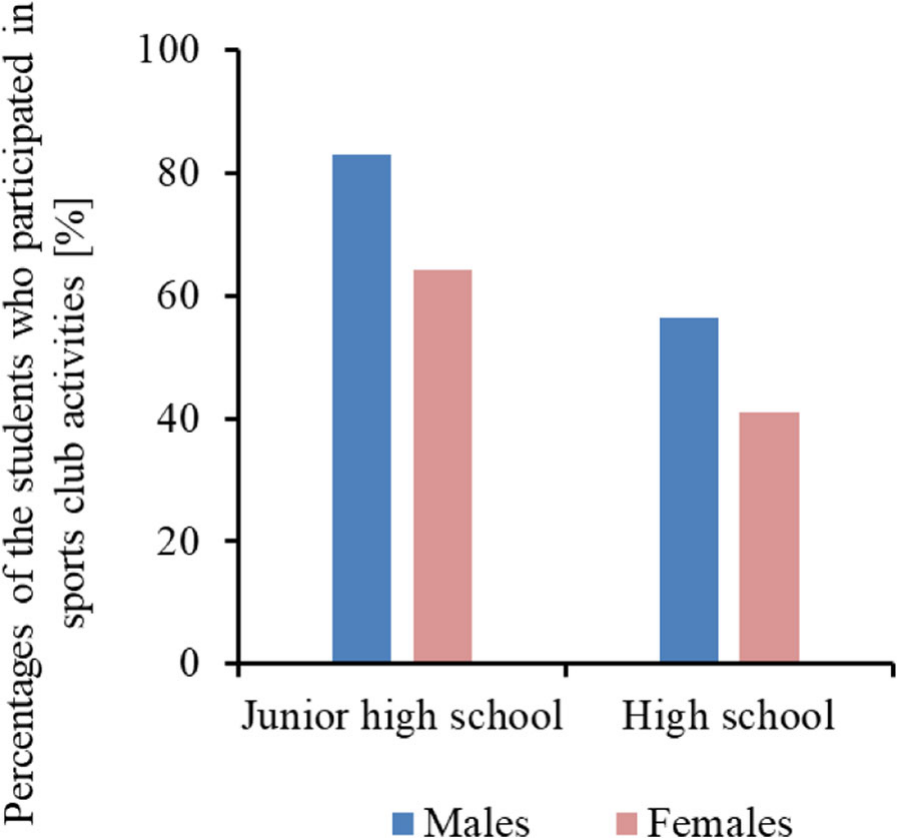
Percentages of the students who participated in sports club activities in Japan. Data from the Ministry of Education, Culture, Sports, Science and Technology [34]

In contrast to adults, the costs of wet compresses prescribed to people aged 19 years or younger were consistently higher among male patients than among female patients (Fig. 2). The appearance of the bar graphs depicting wet compress cost by age and sex was similar to that of the percentages of the students who participated in sports club activities, as shown in Fig. 4. Boys were more physically active than girls in their teenage years. The prescription of wet compress to young people might be because of athletic injuries, although this explanation is still at the stage of hypothesis.

The positive associations of the wet compress cost per resident with the number of orthopedists and rehabilitation physicians per 100,000 residents may suggest that larger numbers of orthopedists and rehabilitation physicians increase the spatial density of these physicians and improve patients’ access to them. Another possible partial explanation is physician-induced demand. To our knowledge, this is the first study to reveal these associations. These findings are significant in real-world medicine because they may suggest a way to reduce Japan’s increasing healthcare costs.

The strengths of the present study include the use of the highly comprehensive nationwide insurance claims data. Because the possibility of sampling error was excluded, we are confident about the robustness of the results. Furthermore, the second version of the “NDB Open Data Japan” included information regarding the top 100 products in terms of the number of prescriptions in each therapeutic classification. This is the update’s largest improvement compared with the first version of the “NDB Open Data Japan,” which only included information covering the top 30 products. This improvement enabled us to avoid underestimation. Several limitations of the study also warrant mention. Although the NDB is a nationwide health insurance claims database, workers’ accident compensation insurance, compulsory automobile liability insurance, welfare, and private expenses (e.g., over-the-counter drugs) were not covered as of 2017 [11]. There is no guarantee that all of the wet compresses prescribed over the year were actually used by the patients. A restriction on the number of wet compresses prescribed per patient per visit was introduced in 2016 [35]. Further observation of wet compress cost over time is necessary. Additionally, because the study design of the association analysis was ecological, we cannot be certain that there were no unadjusted confounders.

## Conclusions

The annual pharmaceutical cost of wet compresses prescribed in Japan in fiscal year 2015 totaled 149.0 billion JPY (1.18 billion euros; 1.33 billion USD). The number of orthopedists and rehabilitation physicians per 100,000 residents may be independent predictors of Japan’s wet compress cost. Our findings will facilitate appropriate budget allocation and preventive measures such as quadriceps strengthening exercises and weight control.

## Data Availability

Only administrative data were used in this study. All data sheets are downloadable from governmental websites. Their URLs are shown in the References.

## Abbreviations

DPC: Diagnosis Procedure Combination
JPY: Japanese yen
NDB: National Database of Health Insurance Claims and Specific Health Checkups of Japan
SAS: Statistical Analysis System
USD: United States dollars
